# Supplementary material for: Systematic visualisation of molecular QTLs reveals variant mechanisms at GWAS loci
Source: bioRxiv. 2023 Apr 7:2023.04.06.535816. Preprint. [Version 1] doi: 10.1101/2023.04.06.535816 (PMC10104061; doi:10.1101/2023.04.06.535816)
Supplement: Supplement 2 [file NIHPP2023.04.06.535816v1-supplement-2.pdf]

Supplementary Note

Reference mapping bias is known to induce false positive associations in splicing and allele specific expression analysis (Kumasaka et al., 2016; Li et al., 2018; van de Geijn et al., 2015). A tool often used to correct for reference mapping bias is WASP (van de Geijn et al., 2015), which has also been included in the STAR (Dobin et al., 2013) RNA-seq short read aligner. Although the eQTL Catalogue uses HISAT2 (Kim et al., 2019) to perform RNA-seq read alignment, we did consider the option to switch to STAR to use WASP read filtering. However, after initial benchmarks we opted against it. First, we found that WASP was very conservative and filtered out a large proportion of reads form exonic regions. As a result, many well-known true positive splicing QTLs were no longer detected and the QTL read coverage plots became noisy due to the large number of filtered reads. Secondly, as implemented in STAR, WASP was only able to account for single nucleotide variants and did not consider short insertions or deletions that have even large potential to cause reference mapping bias. Finally, our transcript usage and txrevise quantification uses Salmon (Patro et al., 2017) to pseudoalign reads directly to the transcriptome and is thus not compatible with WASP. For example, two of the three suspected reference mapping bias cases (*DHCR7* and *NUDT9*) were detected in Salmon transcript usage analysis. Finally, switching to STAR+WASP would have significantly increased the runtime of our RNA-seq quantification workflow which already took over two months at the University of Tartu High Performance Computing Center. For these reasons we decided against directly correcting for reference mapping bias in the QTL mapping process. Instead, we opted to provide access to pre-generated QTL coverage plots that can be used to visually detect strong cases of reference mapping bias.

bioRxiv preprint doi: https://doi.org/10.1101/2023.04.06.535816; this version posted April 7, 2023. The copyright holder for this preprint (which was not certified by peer review) is the author/funder, who has granted bioRxiv a license to display the preprint in perpetuity. It is made available under aCC-BY 4.0 International license.

Supplementary Note 2

Funding statements for the new studies included in the eQTL Catalogue.

CommonMind. Bio-samples and/or data for this publication were obtained from NIMH Repository & Genomics Resource, a centralized national biorepository for genetic studies of psychiatric disorders. Data were generated as part of the CommonMind Consortium supported by funding from Takeda Pharmaceuticals Company Limited, F. Hoffman-La Roche Ltd and NIH grants R01MH085542, R01MH093725, P50MH066392, P50MH080405, R01MH097276, RO1-MH-075916, P50M096891, P50MH084053S1, R37MH057881, AG02219, AG05138, MH06692, R01MH110921, R01MH109677, R01MH109897, U01MH103392, and contract HHSN271201300031C through IRP NIMH. Brain tissue for the study was obtained from the following brain bank collections: the Mount Sinai NIH Brain and Tissue Repository, the University of Pennsylvania Alzheimer's Disease Core Center, the University of Pittsburgh NeuroBioBank and Brain and Tissue Repositories, and the NIMH Human Brain Collection Core. CMC Leadership: Panos Roussos, Joseph Buxbaum, Andrew Chess, Schahram Akbarian, Vahram Haroutunian (Icahn School of Medicine at Mount Sinai), Bernie Devlin, David Lewis (University of Pittsburgh), Raquel Gur, Chang-Gyu Hahn (University of Pennsylvania), Enrico Domenici (University of Trento), Mette A. Peters, Solveig Sieberts (Sage Bionetworks), Thomas Lehner, Stefano Marenco, Barbara K. Lipska (NIMH).

CAP. The dataset used for the analyses described in this manuscript was obtained from the Cholesterol and Pharmacogenetics (CAP) study through dbGAP (phs000481.v3.p2). Funding support for the generation of this dataset was provided by National Heart, Lung, Blood Institute (NHLBI) grant U01 HL69757. The manuscript was not prepared in collaboration with CAP investigators and does not necessarily reflect the opinions or views of CAP investigators or NHLBI.

Peng_2018. This work was supported by the National Institutes of Health [NIH-NIMH R01MH094609, NIH-NIEHS R01ES022223, NIH-NIEHS PO1ES022832, NIH-NIEHS R24ES028507, NIH-NIEHS R21ES028226, and NIH-NIEHS R01ES025145. A complete description of the cohort can be found in: Appleton AA, Murphy MA, Koestler DC, Lesseur C, Paquette AG, Padbury JF, Lester BM, and Marsit CJ. Prenatal Programming of Infant Neurobehavior in a Healthy Population. Paediatr Perinat Epidemiol 2016, 30(4): 367-75.

PhLiPS. This work was supported by grant 5U01HG006398.

iPSCORE. This work was supported in part by a California Institute for Regenerative Medicine (CIRM) grant GC1R-06673 and NIH grants EY021237, HG008118, HL107442, DK105541 and DK112155. iPSC RNA-seq was performed at the UCSD IGM Genomics Center with support from NIH grant P30 CA023100.

Bossini-Castillo_2019. This research was funded by the Wellcome Trust (grant number WT206194). L.B.-C. was supported by the MRC Skills Development Fellowship (MR/N014995/1).

bioRxiv preprint doi: https://doi.org/10.1101/2023.04.06.535816; this version posted April 7, 2023. The copyright holder for this preprint (which was not certified by peer review) is the author/funder, who has granted bioRxiv a license to display the preprint in perpetuity. It is made available under aCC-BY 4.0 International license.

Steinberg_2020. This work was funded by the Wellcome Trust (206194). M.J.C. was funded through a Medical Research Council Centre for Integrated Research into Musculoskeletal Ageing grant (148985). R.A.B. and the Human Research Tissue Bank are supported by the NIHR Cambridge Biomedical Research Centre. J.H.D.B. and G.R.W. are funded by a Wellcome Trust Strategic Award (101123), a Wellcome Trust Joint Investigator Award (110140 and 110141) and a European Commission Horizon 2020 Grant (666869, THYRAGE). A.W.M. receives funding from Versus Arthritis; Tissue Engineering and Regenerative Therapies Centre (21156).

Young_2019. R.F. was supported by funding from the UK Multiple Sclerosis Society (MS50), the Adelson Medical Research Foundation and a core support grant from the Wellcome Trust and MRC to the Wellcome Trust-Medical Research Council Cambridge Stem Cell Institute (203151/Z/16/Z). A.Y. is supported by a Wellcome Trust Clinicians PhD Fellowship (RRZD/029). All data for this study were generated under Open targets project OTAR039. N.K. and D.J.G. were funded by the Wellcome Trust grant WT206194.

Gilchrist_2021. C.K. was supported by Wellcome Trust Investigator Award [204969/Z/16/Z], NIHR Oxford Biomedical Research Centre and Chinese Academy of Medical Sciences (CAMS) Innovation 537 Fund for Medical Science (grant number: 2018-I2M-2-002), Wellcome Trust Grants 090532/Z/09/Z and 203141/Z/16/Z to core facilities Wellcome Centre for Human Genetics, Oxford Biomedical Research Computing (BMRC) facility, a joint development between the Wellcome Centre for Human Genetics and the Big Data Institute supported by Health Data Research UK and the NIHR Oxford Biomedical Research Centre. The study was funded by Wellcome Trust Intermediate Clinical Fellowship to B.P.F. (no. 201488/Z/16/Z). J.J.G. is funded by a National Institute for Health Research (NIHR) Clinical Lectureship.

Braineac2. Mina Ryten, David Zhang, and Karishma D’Sa were supported by the UK Medical Research Council (MRC) through the award of Tenure-track Clinician Scientist Fellowship to Mina Ryten (MR/N008324/1). Sebastian Guelfi was supported by Alzheimer’s Research UK through the award of a PhD Fellowship (ARUK-PhD2014-16). Regina Reynolds was supported through the award of a Leonard Wolfson Doctoral Training Fellowship in Neurodegeneration. All RNA sequencing data performed as part of this study were generated by the commercial company AROS Applied Biotechnology A/S (Denmark).

bioRxiv preprint doi: https://doi.org/10.1101/2023.04.06.535816; this version posted April 7, 2023. The copyright holder for this preprint (which was not certified by peer review) is the author/funder, who has granted bioRxiv a license to display the preprint in perpetuity. It is made available under aCC-BY 4.0 International license.

Supplementary figures

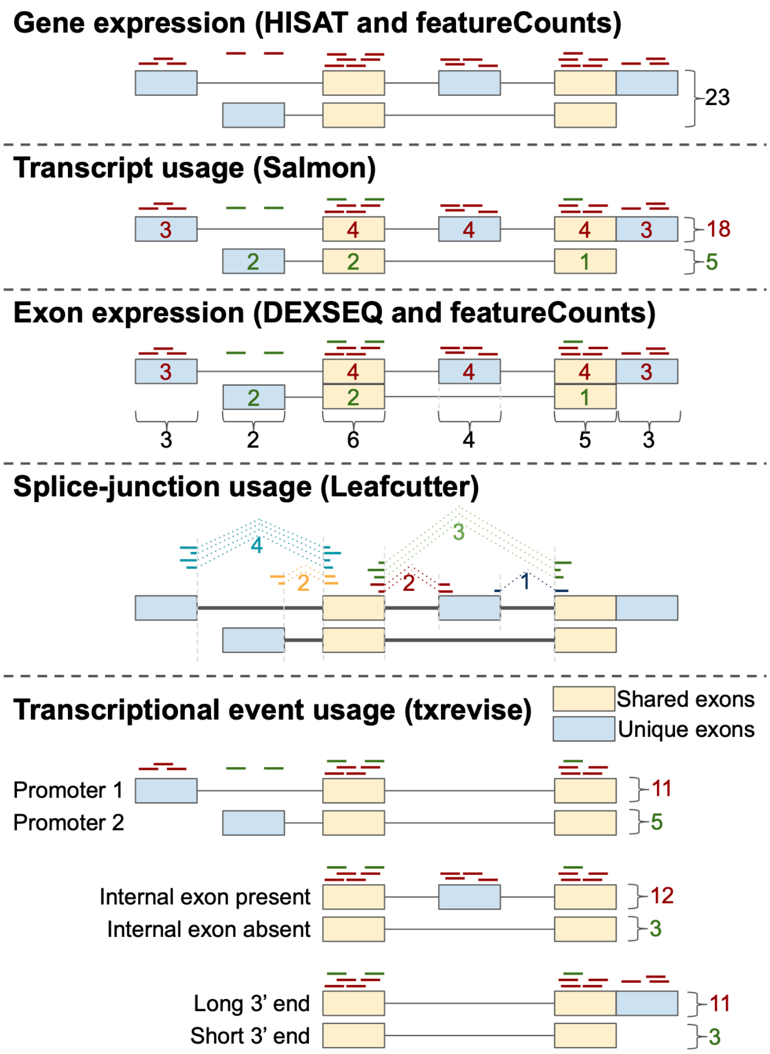

Supplementary Figure 1. Overview of the five molecular trait quantification methods used by the eQTL Catalogue. Gene expression was quantified by counting the total number of reads overlapping annotated exons of the gene. Transcript usage was estimated with Salmon. Exon expression was estimated by counting the number of reads overlapping each exon. Splice-junction usage was quantified with Leafcutter. Txrevise was used to estimate the expression levels of three types of transcriptional events (promoter usage, splicing and 3′ end usage).

bioRxiv preprint doi: https://doi.org/10.1101/2023.04.06.535816; this version posted April 7, 2023. The copyright holder for this preprint (which was not certified by peer review) is the author/funder, who has granted bioRxiv a license to display the preprint in perpetuity. It is made available under aCC-BY 4.0 International license.

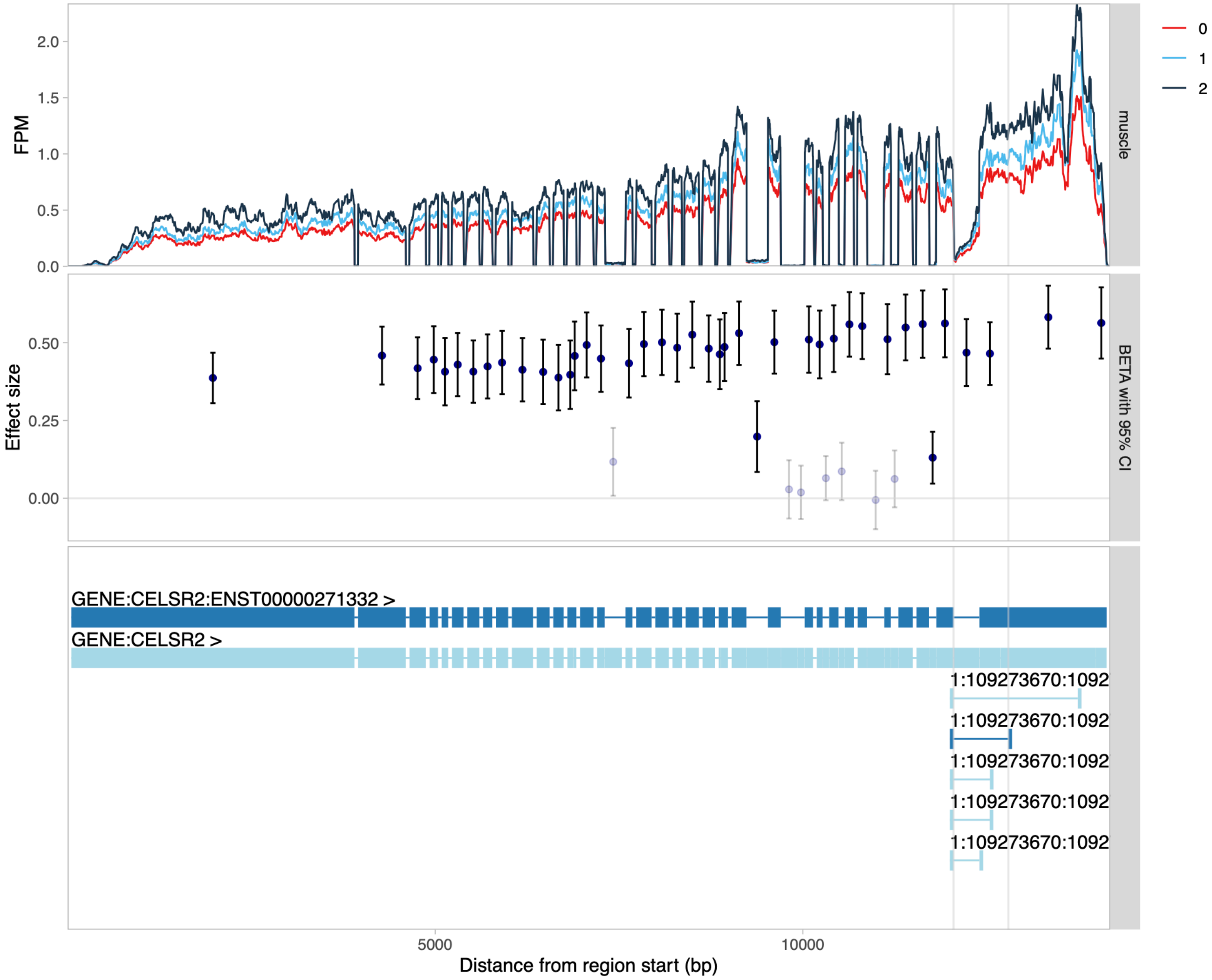

Supplementary Figure 2. QTL coverage plot for *CELSR2* gene stratified by the lead Leafcutter QTL variant (chr1_109274241_T_TC) in the GTEx muscle tissue. The observed association at junction reads in the 3’ end of the gene is likely a consequence of the strong eQTL effect at this locus rather than the primary mechanism driving complex trait association.

bioRxiv preprint doi: https://doi.org/10.1101/2023.04.06.535816; this version posted April 7, 2023. The copyright holder for this preprint (which was not certified by peer review) is the author/funder, who has granted bioRxiv a license to display the preprint in perpetuity. It is made available under aCC-BY 4.0 International license.

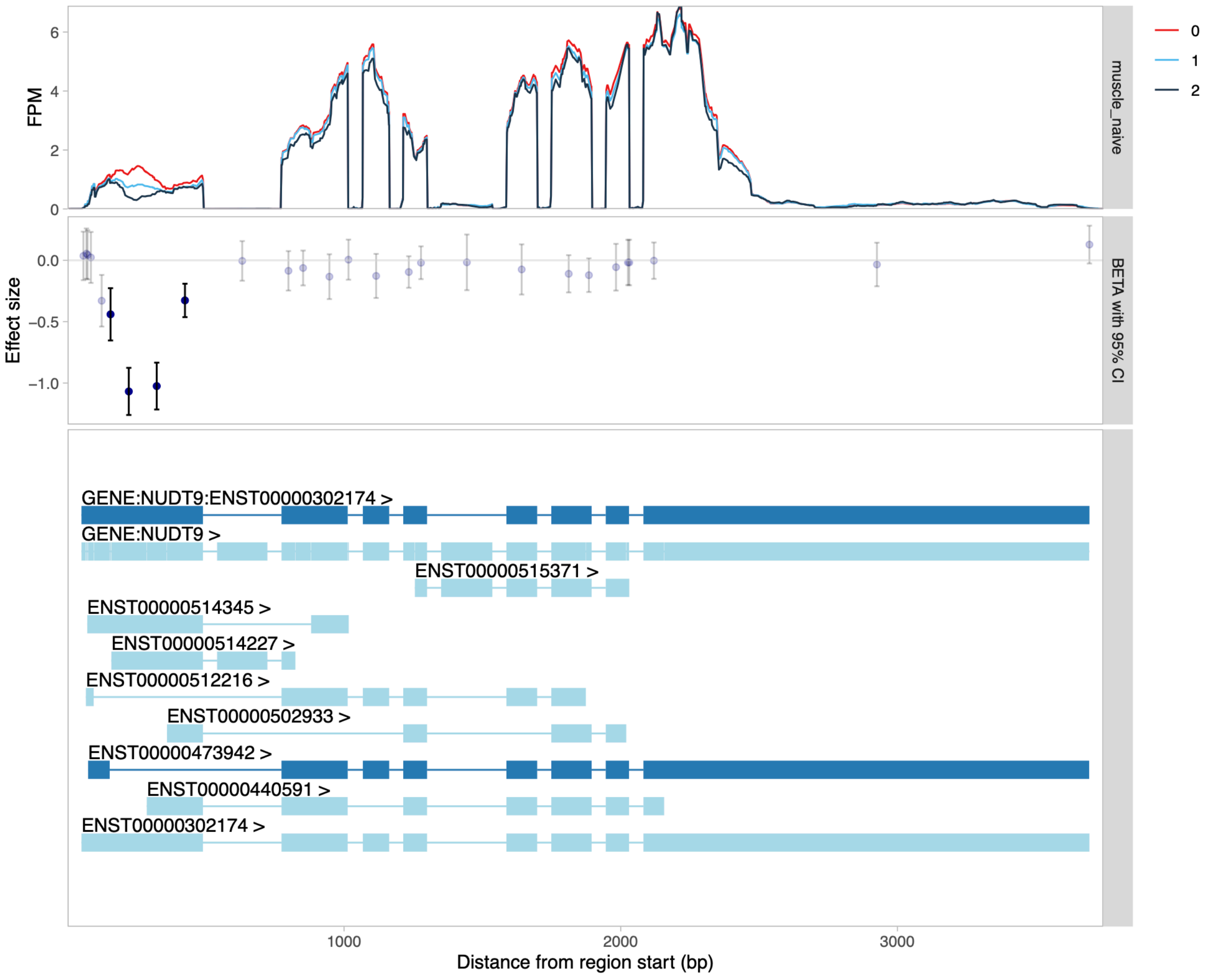

Supplementary Figure 3. QTL coverage plot for *NUDT9* stratified by the genotype of the lead transcript usage (tx) QTL variant (chr4_87380254_C_T) in the FUSION (Taylor et al., 2019) muscle tissue. The 'bulge' in read coverage observed at the 5' end of the gene suggests potential reference mapping bias.

bioRxiv preprint doi: https://doi.org/10.1101/2023.04.06.535816; this version posted April 7, 2023. The copyright holder for this preprint (which was not certified by peer review) is the author/funder, who has granted bioRxiv a license to display the preprint in perpetuity. It is made available under aCC-BY 4.0 International license.

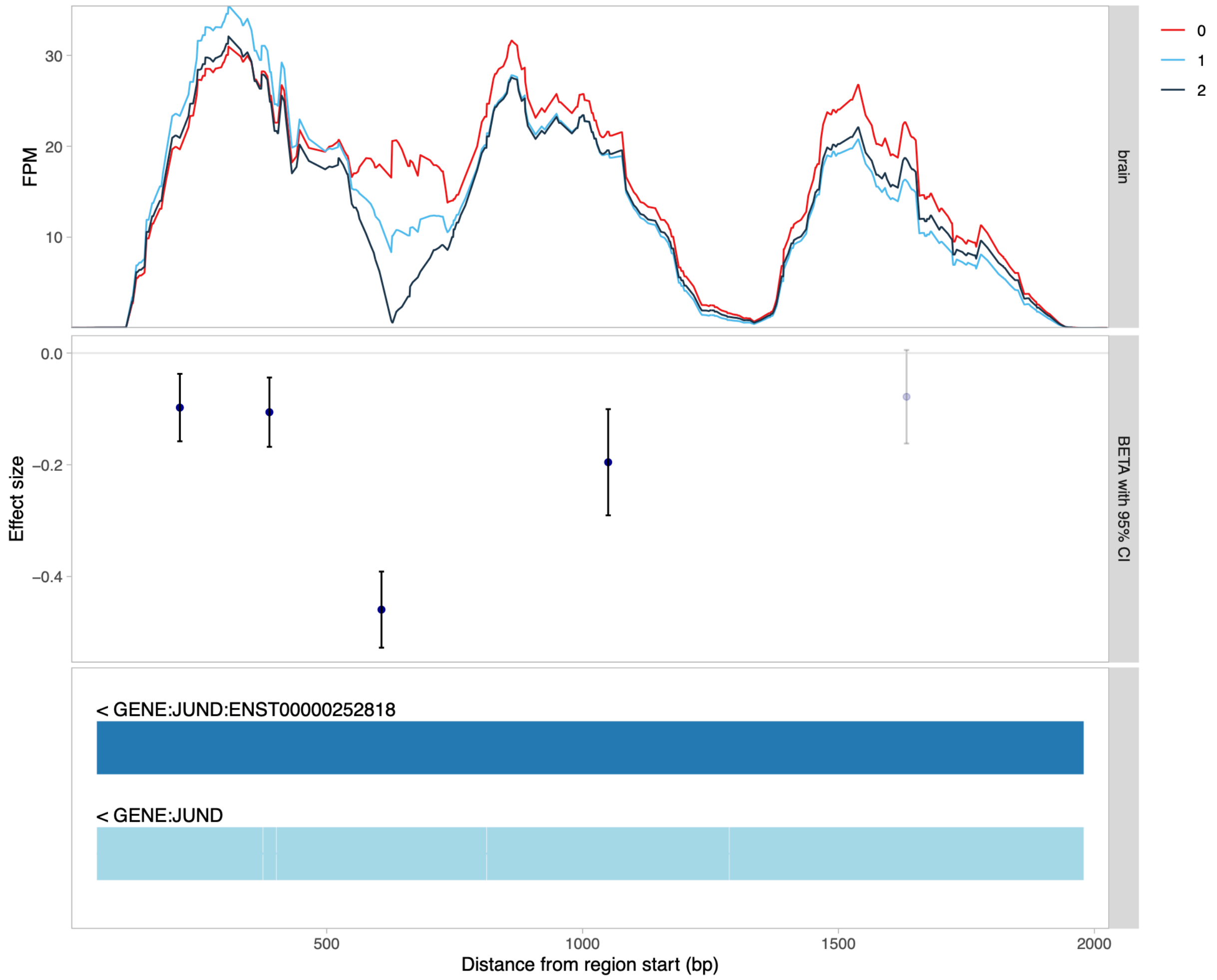

Supplementary Figure 4. QTL coverage plot for *JUND* stratified by the genotype of the lead gene expression (ge) QTL variant (chr19_18287220_A_C) in the BrainSeq (Jaffe et al., 2018) dataset. The drop in read coverage in the middle of the exon suggests potential reference mapping bias.

bioRxiv preprint doi: https://doi.org/10.1101/2023.04.06.535816; this version posted April 7, 2023. The copyright holder for this preprint (which was not certified by peer review) is the author/funder, who has granted bioRxiv a license to display the preprint in perpetuity. It is made available under aCC-BY 4.0 International license.

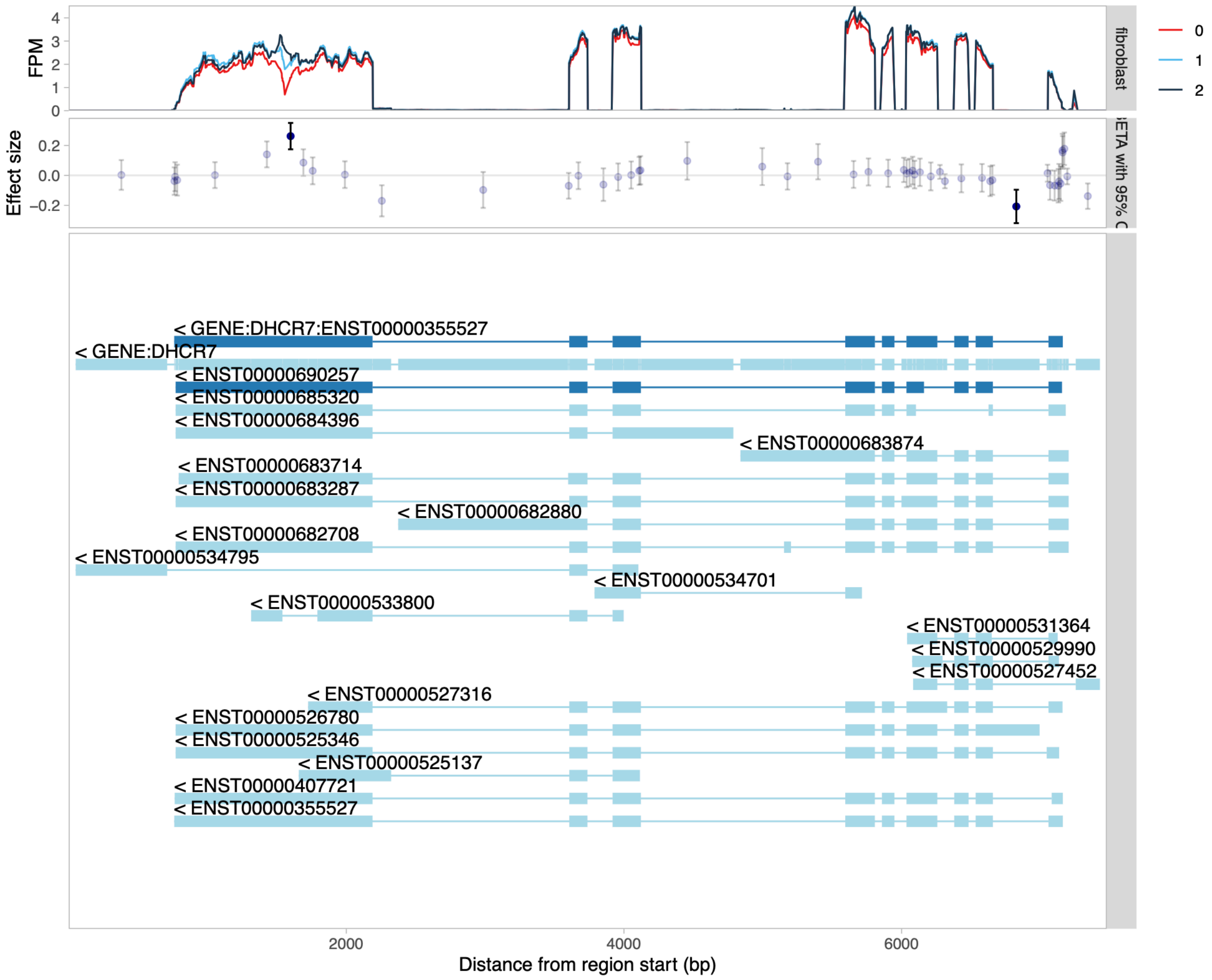

Supplementary Figure 5. QTL coverage plot for *DHCR7* stratified by the genotype of the lead transcript usage (tx) QTL variant (chr11_71458997_T_C) in the GTEx fibroblast dataset. The 'bulge' in read coverage observed at the middle of the last exon of the MANE Select transcript (ENST00000355527) suggests that the association is driven by reference mapping bias.
